# Matrix-assisted laser desorption/ionization time-of-flight mass spectrometry (MALDI-TOF MS) shows adaptation of grass pollen composition

**DOI:** 10.1038/s41598-018-34800-1

**Published:** 2018-11-08

**Authors:** Sabrina Diehn, Boris Zimmermann, Murat Bağcıoğlu, Stephan Seifert, Achim Kohler, Mikael Ohlson, Siri Fjellheim, Steffen Weidner, Janina Kneipp

**Affiliations:** 1 Department of Chemistry, Humboldt-Universität zu Berlin, Brook-Taylor-Straße 2, 12489 Berlin, Germany; 2 BAM Federal Institute for Materials Research and Testing, Richard-Willstätter-Straße 11, 12489 Berlin, Germany; 3 Faculty of Science and Technology, Norwegian University of Life Sciences, 1432 Ås, Norway; 4 Faculty of Environmental Sciences and Natural Resource Management, Norwegian University of Life Sciences, 1432 Ås, Norway; 5 Faculty of Biosciences, Norwegian University of Life Sciences, 1432 Ås, Norway

## Abstract

MALDI time-of-flight mass spectrometry (MALDI-TOF MS) has become a widely used tool for the classification of biological samples. The complex chemical composition of pollen grains leads to highly specific, fingerprint-like mass spectra, with respect to the pollen species. Beyond the species-specific composition, the variances in pollen chemistry can be hierarchically structured, including the level of different populations, of environmental conditions or different genotypes. We demonstrate here the sensitivity of MALDI-TOF MS regarding the adaption of the chemical composition of three *Poaceae* (grass) pollen for different populations of parent plants by analyzing the mass spectra with partial least squares discriminant analysis (PLS-DA) and principal component analysis (PCA). Thereby, variances in species, population and specific growth conditions of the plants were observed simultaneously. In particular, the chemical pattern revealed by the MALDI spectra enabled discrimination of the different populations of one species. Specifically, the role of environmental changes and their effect on the pollen chemistry of three different grass species is discussed. Analysis of the group formation within the respective populations showed a varying influence of plant genotype on the classification, depending on the species, and permits conclusions regarding the respective rigidity or plasticity towards environmental changes.

## Introduction

Matrix-assisted laser desorption/ionization (MALDI) is a common soft ionization method for mass spectrometry (MS). Since the molecules show relatively low fragmentation in MALDI, the method is ideal for the investigation of large, intact molecules such as lipids^1^ and proteins^2,3^. Hence, MALDI time-of-flight mass spectrometry (MALDI-TOF MS) has become a suitable tool for the classification of complex biological samples, such as bacteria^2,4–9^, fungi^10,11^, and tissue samples of animals^12,13^ and plants^14–16^. In MALDI-TOF MS, the contributions from the mass-to-charge ratios of all the different molecular species contained in a complex sample leads to a highly specific, fingerprint-like spectrum. This utilization of the full and complex molecular constitution is similar to vibrational spectroscopic methods, which have been used for fingerprinting of biological samples for several decades^17–20^. However, MALDI-TOF-MS provides different selectivity that is brought about by specific sample preparation and the extraction of analyzed molecules. Therefore, the MALDI-TOF MS approach can be considered complementary to vibrational spectroscopy for many analytical problems.

We and others have shown that MALDI-TOF MS can help to investigate purified pollen allergenic molecules^21–24^ and to classify whole pollen of different plant species^25–28^. In combination with multivariate tools such as hierarchical cluster analysis (HCA) or principal component analysis (PCA), MALDI-TOF MS enables a robust discrimination between pollen species^27^. Moreover, high throughput identification can be achieved using sticky carbon tape for pollen trapping and fixation^26^.

Nevertheless, the basis of the molecular classification of pollen is understood best for the vibrational spectroscopic characterization approaches so far^29,30^. Raman spectroscopy^31,32^, as well as infrared (IR) spectroscopy^33,34^ have been used for years to investigate the chemical composition of pollen. There, a hierarchical structure of classification data, ranging from phylogenetic relationships^32–39^, over altered environmental conditions^40–47^, to subtle physiological changes^48,49^ is discussed. MALDI-TOF MS would add complementary information about the pollen composition and enable a more complete insight to the chemical composition of pollen grains.

Recently, systematic classification studies by IR spectroscopy have been conducted in a large grass pollen sample set that comprises three different levels of discrimination (i) different species, (ii) different populations of the same species, and (iii) different growth conditions provided to the plants of a specific species and population^47^. This experimental design enabled the identification of varied adaptation of the pollen chemistry to environmental influences during the growth of the plants for different populations, as well as a discussion of their phenotypic plasticity.

Here, we study the aforementioned pollen samples from three grass species, namely *Poa alpina, Anthoxanthum odoratum*, and *Festuca ovina*^47^, in order to assess whether the MS fingerprint can be used to detect variation in pollen chemistry beyond the identification of plant species, that has been demonstrated so far with the MALDI-TOF MS approach^25,27^. In particular, we are interested whether differences in the chemical composition of pollen within a species and a population, respectively, can become evident in the MALDI-TOF spectra so that different samples can be discriminated. A discrimination between pollen samples obtained from parent plants grown at different temperature and nutrient conditions would give us an information about phenotypic plasticity, e.g., very similar pollen spectra obtained from pollen samples grown at very different conditions would indicate high rigidity of the phenotypes. The influence of growth conditions on pollen grain chemical composition is of interest, since pollen chemistry is the direct result of a complex coordination of both gametophytic (i.e., pollen grain) and sporophytic (i.e., parent plant) metabolism^50^. Chemical differences in pollen composition are a strong indication of differences in pollen physiology, which could lead to differences in siring success. For example, recent studies have shown that temperature can have a large effect on pollen germination and pollen tube growth^44,45^. In our study, MALDI-TOF MS was applied in combination with partial least squares discriminant analysis (PLS-DA) to identify potential variances between species, populations, and growth conditions. The studied set of 272 samples has been previously thoroughly characterized by infrared spectroscopy, biogeography, and pollen phenology^47^. As shown in the analysis here, the MALDI-TOF spectral information can result in chemical differentiation in some, but not in all categories. In particular, while variance between species and populations is detected, the effects of specific growth conditions cannot be observed in all populations and species. For the latter, as shown in an FTIR study with the same samples^47^, vibrational spectra can be used. The results demonstrate that the MS spectra provide information that adds to the molecular fingerprinting of vibrational spectroscopies, and thereby the MALDI-TOF MS approach complements identification of intra-species variation and phenotypical plasticity in pollen.

## Results and Discussion

### Classification of MALDI-TOF MS grass pollen spectra at the levels of species and populations

**Figure 1:**
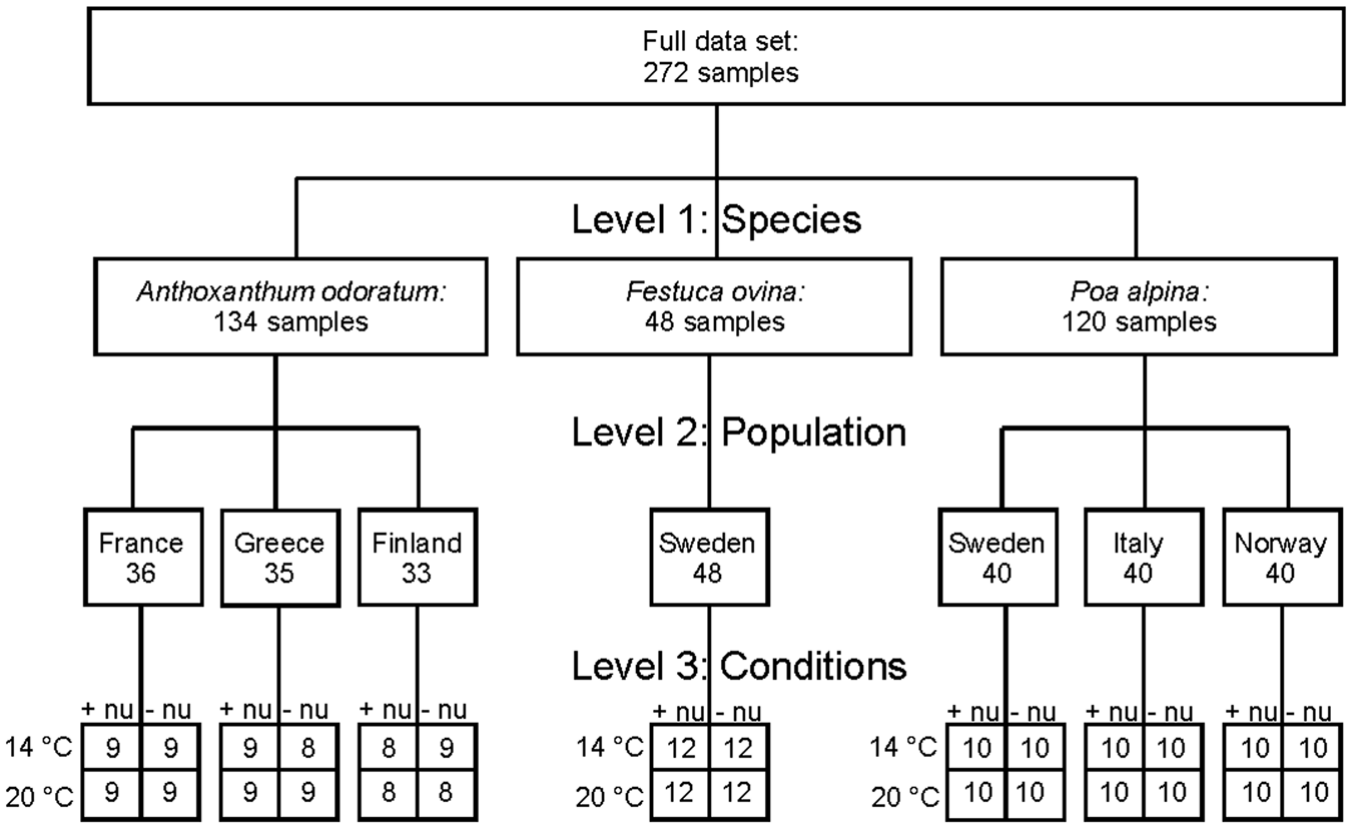
Scheme displaying the numbers of samples (i.e., the number of MALDI-TOF MS spectra obtained) for each level of discrimination. Abbreviations: +nu, additional nutrients, −nu, no additional nutrients.

**Figure 2:**
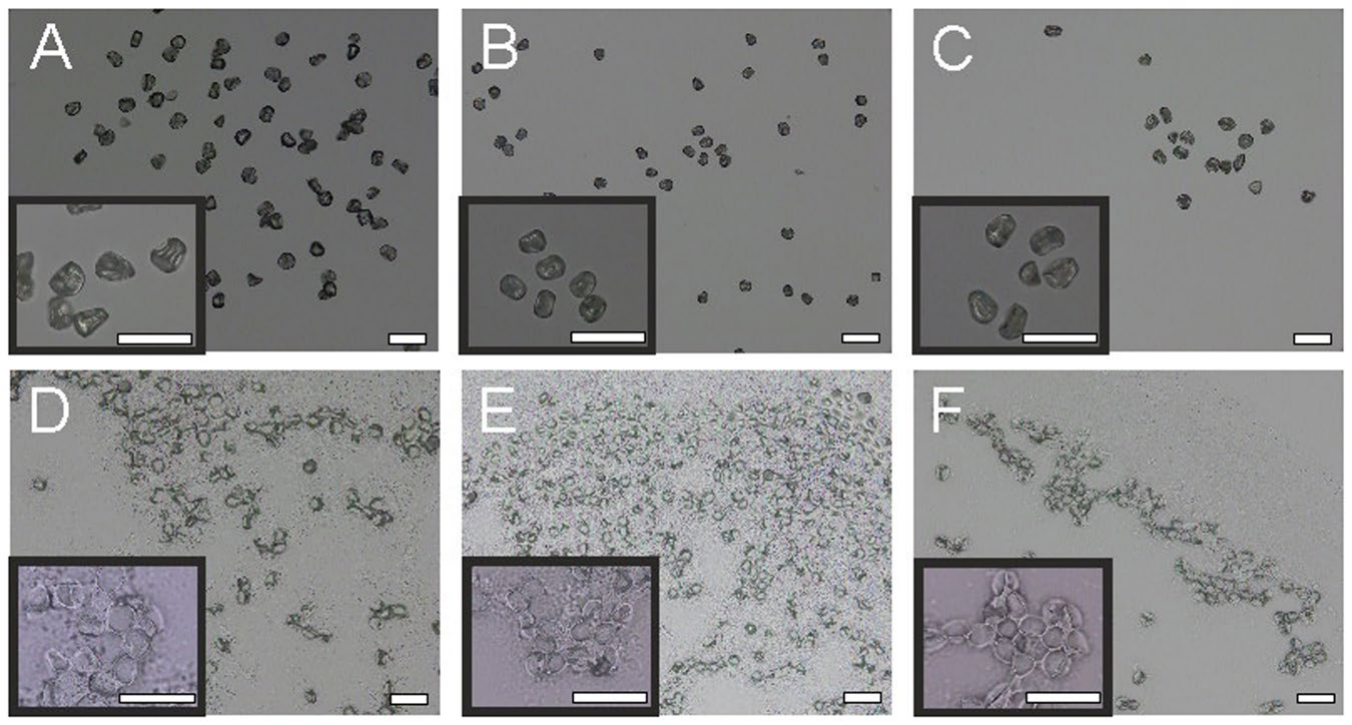
Bright field images of the three different pollen species *A. odoratum* (**A**,**D**)*, F. ovina* (**B**,**E**) and *P. alpina* (**C**,**F**) before (**A**–**C**) and after adding formic acid (**D**–**F**). Images were taken with two different magnifications. Scale bar = 50 µm.

Figure 1 summarizes the number of spectra for each species, population, and growth condition. In our experiment, the two species *Poa alpina* and *Anthoxanthum odoratum* were represented by three different populations each while the samples of *Festuca ovina* pollen originated from only one population (Fig. 1). Each population contained pollen samples obtained under four different growth conditions of the parent plants (Fig. 1). MALDI-TOF mass spectra were collected from a stainless steel target from the acid extract of the pollen. Figure 2 shows bright field images from the pollen of each species before (Fig. 2A–C) and after the treatment with formic acid (Fig. 2D–F). The morphology of the pollen grains does not change during the formic acid treatment. In total, 272 spectra were obtained from the sample set.

**Figure 3:**
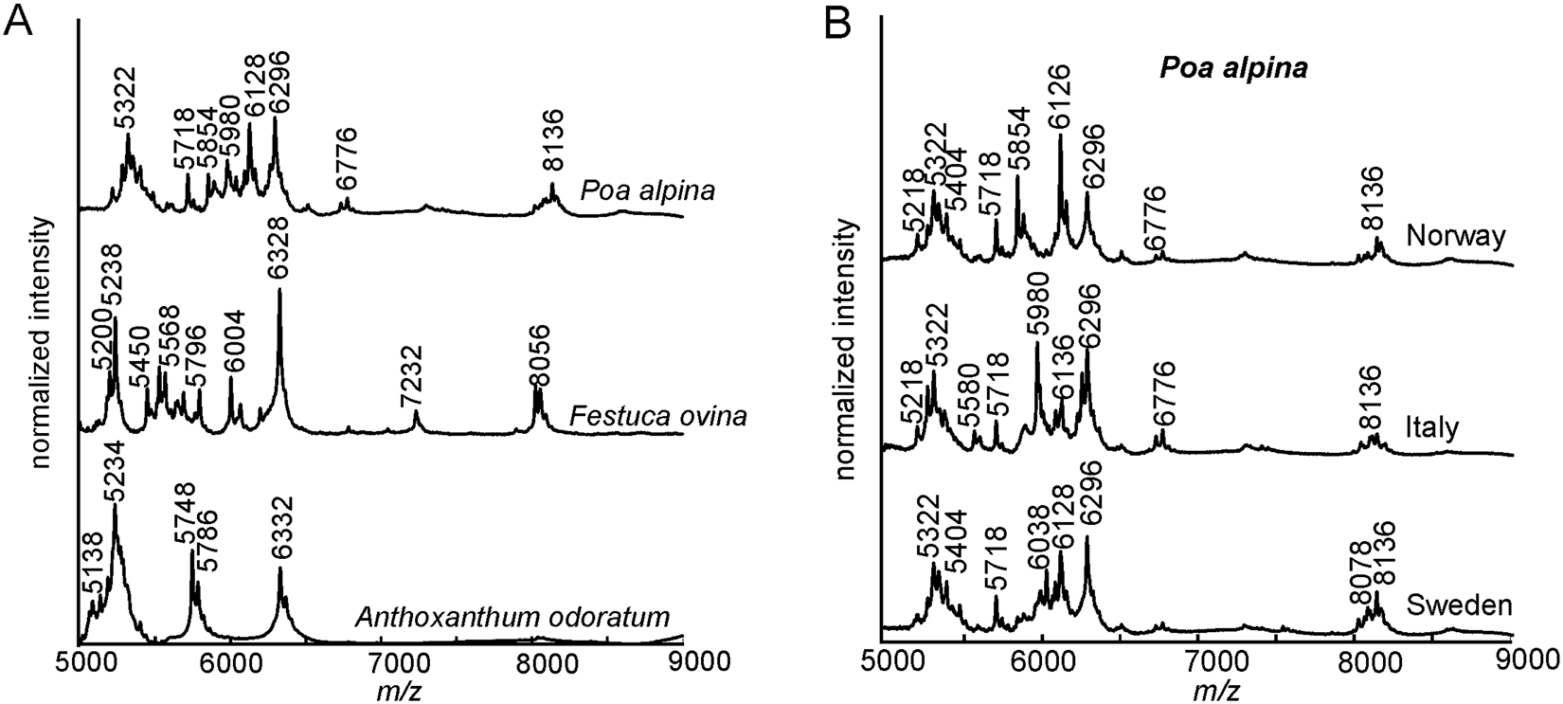
(**A**) MALDI TOF mass spectra of pollen from three different grass species in the range *(m/z)* 5000–9000. (**B**) MALDI-TOF mass spectra for the three different population within *Poa alpina* in the range *(m*/*z)* 5000–9000. All spectra are averages of individual spectra from different samples, with *n* indicating the number of averaged spectra.

In Fig. 3A, an averaged spectrum calculated from single spectra is shown for each pollen species for the mass range *m/z* 5000 to *m/z* 9000. In accordance with previous MALDI measurements covering pollen samples of trees and other plant species^25,27^, the spectra indicate specific characteristics in the chosen mass range that differ greatly for the three species. It is apparent that the peaks above a mass of *m/z* 5000 are highly species-specific. From previous attempts to understand these peak patterns, we associate them here to biopolymers, possibly oligosaccharides^25,27^. The mass spectra of *A. odoratum* pollen grains contain peaks in three distinct mass ranges in the shown region (Fig. 3A bottom trace), while in the case of *F. ovina* and *P. alpina* peaks are distributed over a broader mass range (Fig. 3A upper and middle trace).

Above *m/z* 6500, the amount of peaks in all three spectra is very limited, and the spectra of *A. odoratum* pollen grains do not contain any distinct peaks in this mass range. In the case of *P. alpina* mass spectra, a few peaks around *m/z* 6700, as well as several small peaks around *m/z* 8100 contribute to the species-specific pattern. In the mass spectra of *F. ovina* pollen, several prominent peaks are present above *m/z* 7000, namely at *m/z* 7232, 8024, 8056, and 8092.

As the data in Fig. 3A indicate, MALDI-TOF MS provides highly species-specific spectral patterns, and enables separation of the three pollen species by eye. The large differences in the mass spectral fingerprints of the three Poaceae species are noteworthy, and illustrate the complementarity of mass spectra with other molecular characterization methods for pollen. As shown recently, the FTIR study on the same set of samples revealed that infrared spectra of their pollen provide a highly accurate identification of grass species as well^47^. Previous studies have indicated that the reconstruction of phylogenetic trees of other pollen species using MALDI MS data was more difficult than using vibrational spectroscopies such as FTIR spectroscopy^33^ and Raman scattering^32^. This is due to the fact that vibrational spectroscopic methods probe with less selectivity for different parts of the pollen grains or molecular components than MALDI does, in spite of some effects due to penetration depth and optical cross sections of different IR and Raman approaches^30^. As discussed recently, in MALDI-MS of pollen samples, higher selectivity of probing is inherent due to the preparation procedure and utilization of a matrix, and can in principle also be influenced and varied by varying preparation^26^. Other highly selective spectroscopic investigations of pollen samples that are preceded by extraction steps, such as surface-enhanced Raman scattering, have yielded similar results^36^.

In Fig. 3B, the averaged mass spectra are shown for three populations of *P. alpina*. The chemical patterns differ in relative intensities and in characteristic peaks, e.g., in the mass range between *m/z* 5800 and 6300. In particular, the peak at *m/z* 5854 is prominent in the spectra of the Norwegian population, while it is less present in the other two populations. In comparison to the species-specific differences in Fig. 3A, the distinction of the spectra only by eye is slightly more difficult in Fig. 3B.

For classification of all pollen MALDI-TOF MS spectra from all species and populations, partial least-squares discriminant analysis (PLS-DA) was used, attempting discrimination at the three different levels indicated in Fig. 1. Randomly selected 66% of the respective data was used as training set and the remaining 34% for testing. The success rate (SR) for each level was calculated by PLS-DA to test whether discrimination can be achieved. At the species level, identification of the independent test sets was achieved with SR = 100%, and at the population level with SR = 96%. A much lower success rate (SR = 22%) was obtained for discriminating the 28 classes of spectra from pollen of plants growing at different conditions. For comparison, we also assessed the discrimination by full cross validation. Here, we achieved similar results with SR = 100% for species discrimination, SR = 95% for population discrimination (compare Tables 1 and S1) and SR = 13% for classification of the growth conditions. The latter will be assessed further in the next section for each respective population. Regarding the identification of the species, all mass spectra from the independent test set were identified correctly. In accord with previous findings for other plant species^25–27^, we see here that MALDI-TOF MS is a fast method for discriminating the Poaceae species as well. Pollen from *P. alpina*, *F. ovina* and *A. odoratum* have similar morphology and size^51–53^, which can also be seen in Fig. 2A–C. Therefore, the discrimination in the light microscope is impractical, and fast chemical characterization is very useful. Recently, Zimmermann *et al*. discussed that also the chemical fingerprint obtained by FTIR enables a robust classification for the three grass pollen species^47^. It was shown that the FTIR spectral differences between the grass pollen are strongly dependent on the species-specific ratios of lipids, proteins and carbohydrates in the pollen grains^40,47^. By using mass spectrometry, these large biomolecules, as well as their fragments, can be detected and result in a unique MS pattern with high sensitivity. Nevertheless, conclusions on the molecular basis of the discrimination between the spectra is quite challenging, and a more detailed analysis by, for example, MS/MS-experiments^25^ is needed. In spite of these challenges regarding detailed interpretation, the fingerprints of the biochemical composition of the pollen grains obtained here enable a robust classification according to species from the same family.

**Table 1:** Classification results of an independent test set of pollen MALDI-TOF mass spectra using PLS with 9 latent variables for the classification of seven different populations.

Target class Output class	*A. odoratum* France	*A. odoratum* Greece	*A. odoratum* Finland	*F. ovina* Sweden	*P. alpina* Sweden	*P. alpina* Italy	*P. alpina* Norway
*A. odoratum*, France	10	0	3	0	0	0	0
*A. odoratum*, Greece	0	11	0	0	0	0	0
*A. odoratum*, Finland	1	0	7	0	0	0	0
*F. ovina*, Sweden	0	0	0	22	0	0	0
*P. alpina*, Sweden	0	0	0	0	15	0	0
*P. alpina*, Italy	0	0	0	0	1	12	0
*P. alpina*, Norway	0	0	0	0	0	0	12
Sucess Rate	91%	100%	70%	100%	100%	100%	100%

The numbers in the cells indicate the amount of spectra classified as the respective output class.

As can be seen in Table 1, the discrimination of different populations has high success rates in both *A. odoratum* and *P. alpina*, which are represented by three different populations each (Table 1). In Table 1, the details on the misclassified spectra (4% of the independent test set) are shown. One out of 11 spectra from the French population of *A. odoratum* was classified as Finnish population. In addition, three out of 10 spectra of the Finnish population were misclassified as French population. In the case of the *P. alpina* populations, there is no incorrectly classified spectrum. The success rate of 96% for discrimination of the populations in general indicates that the chemical pattern revealed by MALDI-TOF MS is very specific for this parameter, and that the different populations have developed several phenotypic differences. It should be noted that classification of the same samples at the population level by FTIR spectroscopy has achieved much lower success rates than MALDI-TOF MS, in particular for *A. odoratum* samples^47^. While FTIR measurements indicated within-species variation for the different populations of *P. alpina*, these differences were not present for populations of *A. odoratum*, thus leading to the conclusion that the populations must be very similar^47^. As demonstrated here, using the chemical information provided by MALDI-TOF MS, there is a significant variation of pollen grain chemistry between different populations of both *P. alpina* and *A. odoratum*. Thus, including the information from MALDI-TOF MS in investigations of overall pollen biochemistry will complement spectroscopic characterization, and in particular help to determine phenotypic similarities between different populations in order to assess, e.g., possibilities of gene flow between them.

### Classification of grass pollen MALDI spectra at the level of growth conditions

For the investigation of a potential effect the different growth conditions may have on pollen chemistry, a PLS model was calculated for each population, so that variances between and within species can be excluded. Compared to the discrimination analyses of species and populations, where independent test and validation sets were used, the discrimination analyses regarding growth conditions was based on leave-one-out full cross-validation due to relatively small spectral sets. The within-group variance regarding the different conditions was obtained by using the Kruskal-Wallis test, a nonparametric statistical test based on the ranks of the data. The tests were performed on the score values of the first principal component, and defining the conditions as groups. As a result, the information obtained by PCA is reduced according to the respective distribution of the score values. In this analysis, a low p-value (p < 0.05) indicates, that there is evidence of the score values being similar enough to assume separation of the scores on a significance level of 5%. Table 2 lists the success rates in a leave-one-out full cross-validation (Table 2, third column) and p-values (Table 2, fourth and last column) for each population concerning the two varied environmental conditions, temperature and nutrient content, both, separately, as well as in combination (Table 2, last section). The data sets show good discrimination (SR > 70%) of different temperatures for five populations (Table 2, first section), but only the Norwegian population of *P. alpina* has displayed a significant discrimination (p < 0.05). Only for this population, we therefore conclude a significant influence of the variation in temperature during plant growth on pollen chemistry revealed in the MALDI mass spectra. Looking at the correlation between the MALDI-MS fingerprint and the nutrient conditions (Table 2, second section), two populations show good discrimination, but only the Italian population of *P. alpina* has p < 0.05, indicating that pollen chemistry is influenced by nutrient availability prior to pollination. The results are in good agreement with the results of the FTIR study on the same pollen samples, which has also shown that temperature effects are influencing pollen chemistry to a higher degree than nutrient effects^47^.

**Table 2:** Results obtained by PLS and PCA. Success rates (SR) for classification of the pollen spectra from samples of the four cultivation conditions for full cross validation (full-CV) using PLS with the optimal amount of latent variables, as well as p-values of the first principal component calculated using a Kruskal-Wallis test.

	Pollen species/population	Optimal amount of latent variables	SR full-CV	p-value (1^st^ PC)	p-value (lowest*)
temperature (14 C/20 °C)	*A. odoratum*, France	6	**56%**	0.0665	0.0290, PC9
	*A. odoratum*, Greece	7	**74**%	0.4093	0.0017, PC7
	*A. odoratum*, Finland	8	30%	0.3132	—**
	*F. ovina*, Sweden	11	**77%**	0.5362	—**
	*P. alpina*, Sweden	7	**78%**	0.1762	0.0119, PC7
	*P. alpina*, Italy	7	**80%**	0.0620	0.0149, PC5
	*P. alpina*, Norway	7	**78%**	0.0173	
nutrients (+nu/−nu)	*A. odoratum*, France	7	47%	0.4477	—**
	*A. odoratum*, Greece	7	46%	0.2761	0.0175, PC5
	*A. odoratum*, Finland	7	48%	0.1303	—**
	*F. ovina*, Sweden	12	48%	0.6501	—**
	*P. alpina*, Sweden	8	**65%**	0.8070	0.0284, PC10
	*P. alpina*, Italy	8	**98%**	0.0017	
	*P. alpina*, Norway	8	**83%**	0.1046	0.0038, PC5
all conditions	*A. odoratum*, France	8	42%	0.0226	
	*A. odoratum*, Greece	7	34%	0.5733	0.0485, PC5
	*A. odoratum*, Finland	6	6%	0.13	0.0544, PC3
	*F. ovina*, Sweden	11	33%	0.8928	0.0446, PC9
	*P. alpina*, Sweden	9	35%	0.5412	0.0294, PC7
	*P. alpina*, Italy	7	**63%**	0.0038	
	*P. alpina*, Norway	7	**70%**	0.0047	

Analysis was done for different temperatures, nutrient conditions and the combination of both for each population, respectively (sections top to bottom). *Lowest p-value and respective principal component for the 2^nd^ to 10^th^ PC.

**No significant p-value could be calculated for the investigated PCs.

When trying to discriminate between pollen obtained from plants grown under different conditions, there is also a chance of discriminating between growth temperatures in spite of very similar MALDI fingerprints for pollen obtained from plants grown under different nutrient conditions, or vice versa. Taking into account all four environmental conditions, the two *P. alpina* populations (Italy and Norway), as well as the French *A. odoratum* population display statistically significant correlations of the chemical composition revealed by MALDI-TOF MS of the pollen and the growth conditions of the respective parent plants (Table 2, last section).

As visible from Table 2, many of the success rates for the leave-one-out cross validation (Table 2, third column) are quite low. Nevertheless, in the species *P. alpina*, the discrimination of the mass spectra can be attained with higher success if the two respective parameters, temperature and nutrient conditions, are analyzed separately (Table 2, third column, first two sections). The relatively high success rates for the discrimination with respect to temperature and nutrient conditions in all three populations of *P. alpina* (Table 2, first two sections) are in good agreement with the success rates obtained with the FTIR data, which are also higher in this than in the two other species^47^. As discussed above, MALDI-TOF MS enables the detection of oligosaccharides and other large biomolecules, which could give a fingerprint of the composition of nutrients stored inside the pollen grains. As also revealed by the FTIR study, variation in both temperature and nutrients provided to the parent plant can influence the content of poly- and oligosaccharides and proteins^47^. Further correlative studies using both MALDI and FTIR could provide a more precise interpretation of the MALDI spectra.

**Figure 4:**
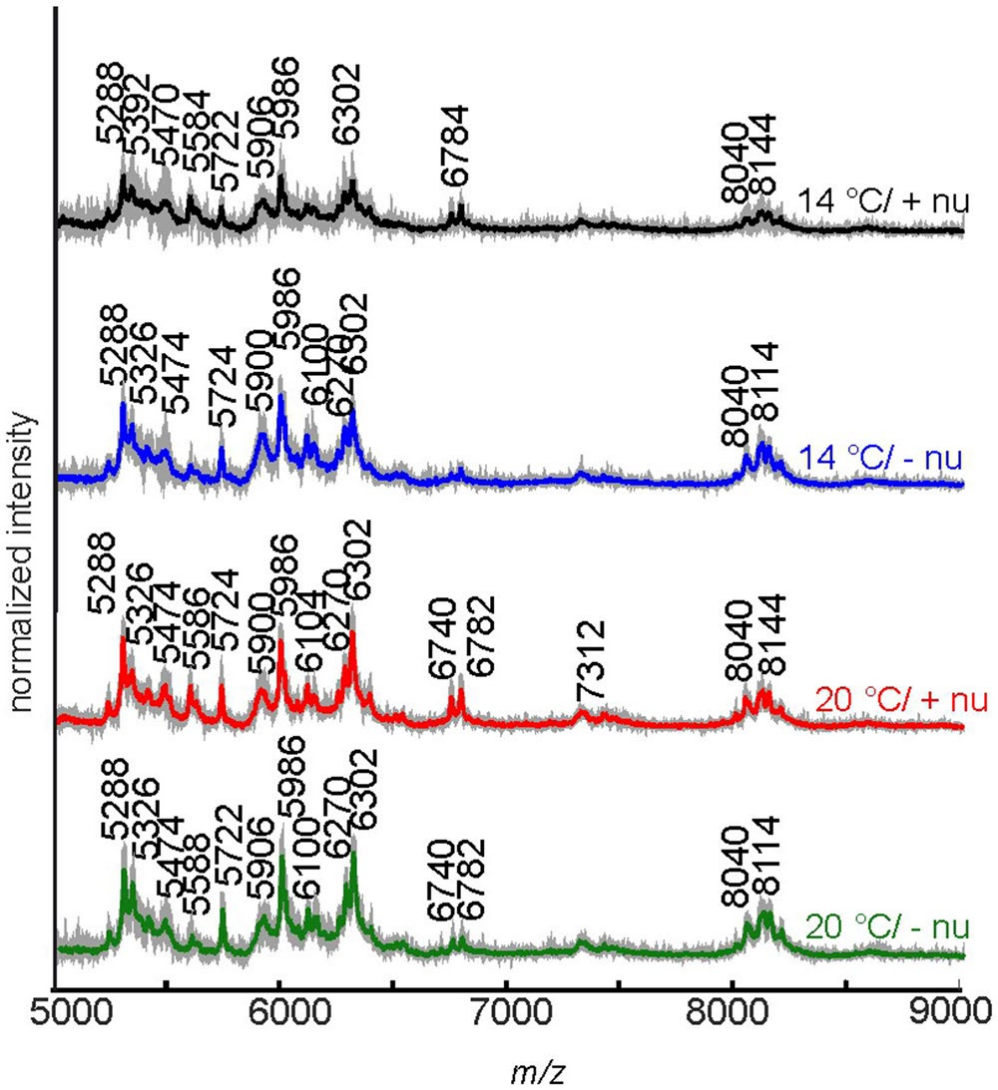
MALDI TOF mass spectra for each of the four cultivation conditions of the plants producing the pollen for the Italian population of *P. alpina*. All four spectra are averages of 10 individual spectra from 10 individual plants; the grey shading indicates the standard deviation. Abbreviations: +nu, additional nutrients, −nu, no additional nutrients.

In Fig. 4, the averaged spectra and the standard deviation indicating the variation for all individual plants of the Italian *P. alpina* population are shown for each of the four growth conditions of the parent plants. The averaged spectra show very similar peak patterns with exceptions in the mass range from *m/z* 5400 to *m/z* 5900, where the peak at *m/z* 5474 is very pronounced in the spectra for pollen of plants grown at 20 °C, while it is less dominant in plants that were grown at 14 °C. As indicated by the high standard deviation, the presence of this peak strongly depends on the investigated individual. As will be discussed below, knowledge about the genotype of the individual plants can enable further discussion about the variation of this and other spectral features, e.g., the peak at *m/z* 5900. The peak at *m/z* 5584 is more prominent in mass spectra from pollen of parent plants treated with additional nutrients.

**Figure 5:**
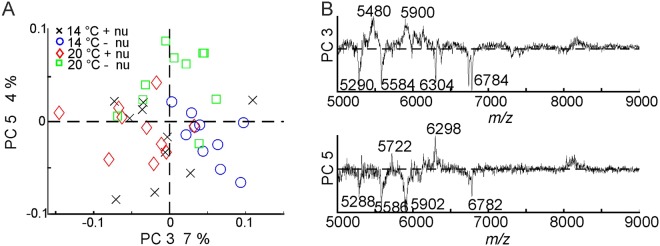
(**A**) Scores plot using PC 3 and PC 5 as a result of a PCA using the mass range m/z 5000–9000 with the individual spectra of the samples obtained from Italian population of *P. alpina* population PAp2. Each of the four cultivation conditions is represented by 10 individual spectra. Abbreviations in the figure legend: +nu, additional nutrients, −nu, no additional nutrients. (**B**) Corresponding loadings of PC3 and PC 5.

To investigate, which variables of the mass spectra cause the influence of the growth conditions on the MALDI-TOF MS spectra, principal component analysis (PCA) was carried out with the mass spectra of all samples from the Italian population of *P. alpina*. Figure 5A shows the scores plot and the loadings for the third and fifth principal component (PC). The third PC is correlated with the different nutrient conditions. In particular, most of the mass spectra of pollen obtained from plants that were growing without additional nutrients (Fig. 5A, circles and squares) have positive score values for the third PC, while most of the mass spectra of pollen obtained from plants that were growing at 20 °C and additional nutrients (Fig. 5A, diamonds) have negative values for the third PC. As can be seen in the corresponding loadings spectrum in Fig. 5B, the third PC, which explains 7% of the total variance, is strongly influenced by signals at *m/z* 5290, 5480, 5584, 5900, 6304, and two signals around *m/z* 6780. The signals at *m/z* 5290, 5584, 6304 and the two signals around *m/z* 6780 have negative values, indicating that they are less dominant in the mass spectra of pollen obtained from plants that were growing at 14 °C without additional nutrients (Fig. 5A, circles). In reverse conclusion, these peaks are prominent in the mass spectra of pollen obtained from plants that were growing at 20 °C and in the presence of additional nutrients (Fig. 5A, diamonds).

By the fifth PC, mass spectra of pollen obtained from plants that were growing at 20 °C without additional nutrients (Fig. 5A, squares) can be distinguished from mass spectra of pollen obtained from plants with additional nutrients (Fig. 5A, crosses and diamonds), as well as from pollen obtained from plants that were growing at 14 °C and without additional nutrients (Fig. 5A, circles). The pollen from plants grown at 20 °C without additional nutrients (Fig. 5A, squares) have mostly positive score values for the fifth PC. The fifth PC represents 4% of the entire variance, which is mainly caused by signals at *m/z* 5288, 5586, 5902, 6298 and 6782.

We also analyzed the variance contained in the first and second PC, which should be much higher than those of the third and fifth PC. From the information in the scores and loadings of PC1 and PC2 (not shown here), we find that the greatest variance in the data set of the Italian population of *P. alpina* is caused by differences between the spectra from different individuals rather than growth conditions. The score values of the third or fifth PC result in smaller p values (PC3, p = 0.0012; PC5, p = 0.0026) (cf. Fig. 5). Nevertheless, the low p-value for the distribution of the score values obtained from PC1 (Table 2, last section) still indicates some influence of the different growth conditions.

### Sources of intra-population differences

As the data in Table 2 indicate, the discrimination within each respective population regarding the growth conditions is possible only for very few populations of one of the three species by the MALDI-TOF mass spectra. Nevertheless, intra-population variance, likely to be caused by genotypic variations, is found for all data sets. Using MANOVA on the PCA score values from the first to the tenth PC, we estimated the d-values to assess the dimensionality of the genotype-based groups of spectra in the data of each population. The higher the dimensionality, the more nonrandom variation in the groups can be explained. In this case, a high d-value indicates a higher influence of genotypic variations with respect to other variation in the pollen explained above. The d-values are between 0 and N-1, where N is the amount of observed subgroups, which can be compared to the number of sporophyte genotypes known in the experiment. The first column of Table 3 shows the d-values for each population regarding the genotype-based grouping. In addition, the second column of Table 3 contains the theoretically possible values for the case that within each population, individuals of one genotype would form a subgroup, and these subgroups would form only with respect to genotype (N-1). With the exception of *A. odoratum*, Greece and *A. odoratum*, Finland, pollen were obtained from four clones in each population, one clone for each of the four growth conditions discussed above. In the case of *A. odoratum*, Greece, for one genotype there exist pollen from three clones. In *A. odoratum*, Finland, for each of nine different genotypes, pollen from three clones were studied (see Fig. 1).

**Table 3:** d-values obtained using MANOVA on the score values of PC1 to PC10 on the data set of each population and number of theoretically possible groups (N-1) that could form based on the number of genotypes N that are present in the data of each population, assuming genotype-based cluster formation.

Pollen species/population	d-values	*N-1*
*A. odoratum*, France	4	8
*A. odoratum*, Greece	5	8
*A. odoratum*, Finland	5	8
*F. ovina*, Sweden	8	11
*P. alpina*, Sweden	1	9
*P. alpina*, Italy	0	9
*P. alpina*, Norway	0	9

The d-values indicate the dimensionality of the multivariate vector of the groups for each data set.

For the three *P. alpina* populations, the low d-values indicate that no significant grouping occurs with respect to genotype. In contrast, higher d-values are found for *A. odoratum*. Considering the low correlation according to growth conditions discussed above (Table 2) in the *A. odoratum* populations, we conclude that the genotype could have an influence on group formation here. Obviously, variances based on genotype are competing with the variances for different growth conditions, which explains low correlation with the latter (Table 2). In the population of *F. ovina*, a d-value of 8 is found, corresponding to the significant clustering of nine different genotype-based groups. In this set of samples, twelve genotypes were present. This is in accordance with the low success rate obtained when analyzing classification regarding the growth conditions obtained for this population. Specifically, for the Swedish population of *F. ovina*, the success rate for identifying the four growth conditions was only 33% (compare with Table 2, last section).

The discussion of both, the d-values, revealing possible separation according to genotype, and the success rates of the discrimination between different growth conditions can help to assess phenotypic plasticity of the plants. The different extent to which discrimination of different phenotypes is related to different genotype and to which extent to an adaptation to different growth conditions, reveals the ability of pollen to adapt to altered environmental conditions. Pollen chemistry of *P. alpina* shows high phenotypic plasticity compared to *A. odoratum* and *F. ovina*, with significant variances regarding different growth conditions. On the other hand, different genotypes of *P. alpina*, within each population, do not lead to significantly different phenotypes. This is in agreement with the results obtained by FTIR classification, where the three investigated *P. alpina* populations had been attributed with high phenotypic plasticity^47^. As discussed recently, the phenotypic plasticity of *P. alpina* could be a consequence of the restricted distribution of this species to the alpine and other cold regions, and of the species having a very short pollination period due to the short growth seasons in their habitat^47,54–56^. In contrast, in *A. odoratum* and in particular *F. ovina*, pollen chemical composition revealed by the MALDI-TOF-MS is less affected by the different growth conditions. In general, the results of the MALDI-TOF-MS analysis indicate that class formation is influenced by a specific genotype. This supports the phenotypic rigidity that was discussed for *A. odoratum* and *F. ovina* on the basis of their FTIR data previously^47^. The phenotypic plasticity or rigidity is correlated with the different distribution areas of the grasses.

## Conclusions

Here, we analyzed a hierarchically structured data set of MALDI-TOF mass spectra from grass pollen. The pollen sample set enabled us to investigate the variance of the MALDI-TOF MS data on three levels, containing three species, three populations for two of the species, and four growth conditions for each population, respectively. The number of genotypes in each population was known. Using partial least squares discriminant analysis (PLS-DA), different species were distinguished with high significance. In particular, the chemical pattern revealed by the MALDI spectra enabled discrimination of the different populations of one species. Analysis of the group formation within the respective populations revealed a strong influence of plant genotype for the samples of *Festuca ovina* on mass spectra classification. This indicates a high phenotypic rigidity for the samples from this species. In addition, an influence of plant genotype on mass spectra of pollen was detected for *Anthoxanthum odoratum*, albeit to a lesser extent. These findings are in accord with previous findings obtained by the FTIR spectral patterns^47^. In contrast, high phenotypic plasticity is confirmed for the pollen of *Poa alpina*.

The population-specific influence of several growth conditions on the MALDI fingerprints was investigated using PCA in combination with variance analysis. An influence of the growth condition was only found for some of the populations in one species, while most populations did not show a changed pollen chemical composition with the growth conditions. The phenotypical changes known to occur from vibrational spectroscopic data were not detected by MALDI-TOF MS. On the other hand, comparison with a previous study using FTIR spectra of the same samples^47^ shows that population-related variation in the phenotype within a species is more pronounced by the MALDI-TOF MS spectra than by FTIR spectra. This shows that MALDI-TOF MS and vibrational spectroscopy deliver complementary information on pollen chemistry, and suggests that, ideally, a characterization approach that includes as many chemical data as possible should be used, particularly if the type of classification problem is not identified as clearly as in this study design.

MALDI-TOF MS is a sensitive method for the detection of small biochemical differences within the pollen of one species and provides complementary information to that from other spectroscopic methods. The increase in chemical information content, obtained by combining different spectroscopic methods, could help the future interpretation of the MALDI-TOF MS information, thus leading to further insights regarding plant physiology and biochemistry.

## Methods

### Sample preparation

The three grass species *Poa alpina*, *Anthoxanthum odoratum* and *Festuca ovina*, are distributed in Europe, Asia, North Africa, and North America^57^. *P. alpina* is confined to mountains^58^. For an assessment of the differences in the chemical composition of pollen species, plants of three populations were studied in *A. odoratum* and *P. alpina*. In addition, one population of *F. ovina* was investigated. Seeds of the populations were chosen to cover climatic and geographic variances and were acquired from the Nordic Gene Bank. The populations of *A. odoratum* come from France, Greece, and Finland, and those of *P. alpina* from Sweden, Italy and Norway. The *F. ovina* population was from Sweden (compare with Fig. 1).

Each population had up to 15 individuals of different genotypes grown from seeds in the spring. The individuals grew outside over summer, after which each individual was divided into four clones. The plants were subsequently vernalized for 12 weeks at 4 °C with a day length of 8 hours. After vernalization, both the temperature and the nutrient addition were varied, so that the clones grew under four different conditions. Day length was increased to 16 hours to induce flowering. The plants were grown at high temperature (20 °C) or low temperature (14 °C) in combination with additional nutrients in the irrigation water (+nu) or no additional nutrients in the irrigation water (−nu). Details of the growth experiment can be found in Zimmermann *et al*.^47^. A total of 460 individuals produced inflorescences, of which 272 individuals (i.e., different pollen samples) were selected for the MALDI-TOF-MS analysis. The detailed list of the sample set is given in Fig. 1.

### Preparation of samples for MALDI-TOF-MS and data acquisition

The pollen grain samples were deposited on a MALDI stainless steel target. On each sample, 1 µL of formic acid (90%) was pipetted for extraction. After drying at room temperature, 1 µL of matrix solution (10 mg of α-cyano-4-hydroxycinnamic acid in 1 mL 1:1 acetonitrile/water and 0.1% trifluoroacetic acid) was added to each spot, and the target was left to dry at room temperature. An Autoflex III MALDI-TOF mass spectrometer (Bruker Daltonik, Germany) in positive linear mode and with a 355 nm Smartbeam laser (200 Hz) was used for all measurements. Spectra in the mass range from *m/z* 1000 to 15,000 were recorded for each sample. In order to account for potential heterogeneity within the sample spots, 2000 spectra from four different positions of each spot were accumulated. The data set contains in total 272 spectra, which include one spectrum for each sample, respectively (compare Fig. 1).

### Microscopic images

In order to study the morphology of the pollen grains, as well as the influence of the acid extraction the pollen of the three different species before and after the acid treatment were deposit on glass slides and investigated using a light microscope (Olympus BX23, Hamburg, Germany) with either a 20x or 100x objective (Olympus, Hamburg Germany). The images were recorded with CellSens Standard Software 1.17 (Olympus, Hamburg Germany).

### Data analysis

The obtained spectra were interpolated with a distance of *m/z* 2 between data points in the mass range from *m/z* 5000 to 9000. The distance of *m/z* 2 is chosen, to weight smaller and higher variables equally and so that technical issues from the MALDI-TOF device can be neglected.

A 6 order-polynomial baseline correction was applied before the spectra were vector-normalized.

Classification of the pollen spectra was performed with principal component analysis (PCA) and partial least-squares discriminant analysis (PLS-DA). PCA and PLS-DA were carried out using the mass range *m/z* 5000–9000. The whole data set was organized as a hierarchical tree with the three levels: species, populations, and growth conditions. For the whole data set, three PLS models were calculated to differentiate between species (species level with three groups, see Fig. 1), between populations (population level with seven groups, see Fig. 1) and between different growth conditions for each of the seven populations (growth conditions level with 28 groups, see Fig. 1).

To estimate the appropriate amount of latent variables, the 272 obtained mass spectra for each sample respectively were randomly split into 66% training set and 34% test set, for each of the three models corresponding to the hierarchical levels described above. Using 10-fold cross validation, we determined the optimal amount of latent variables for the calibration of the PLS models by calculating the root mean square error (RMSE) for each latent variable respectively. With each model, calculated with the respective training set and the optimized amount of latent variables, we calculated the success rates for the respective test set. The hierarchical levels were also investigated using full cross validation. Here, the 272 spectra were trained with one PLS model first, to estimate the amount of latent variables using the RMSE. Afterwards, a PLS-DA model for each, discrimination of species, population and growth conditions, respectively, was trained using the whole data set excluding one spectrum. This one spectrum, which was not involved in the training of the model is the test for correct classification. We calculated the success rate permutatively in this way for the whole data set for the 3 different species, the 7 different populations and the 28 groups of the growth condition level (see Fig. 1). The success rate (SR) for each model was calculated by the amount of correctly classified spectra for each group in percent.

For investigation of the growth conditions, three PLS models were calculated for each population, respectively, in order to exclude variances between species and populations, leading to 21 independent PLS models. To estimate the appropriate amount of latent variables, the mass spectra in each data set respectively were trained into one model.

One PLS model was used to build a classification model for groups according to different temperature conditions, one PLS model was established for classification into groups according to different nutrient conditions, and one PLS model was established for classification into classes according to all conditions. We trained each model using the whole data set except one spectrum each and permutated this procedure to apply leave-one-out cross validation (full-CV).

In addition, we investigated the influence of the growth conditions on the within-class variance of each population. PCA score values calculated for each population were tested using Kruskal-Wallis test^59^, using *kruskalwallis*-function in Matlab for each of the three classification problems (temperature, nutrients and the differentiation all conditions). A p-value below 0.05 rejects the null hypothesis that all groups of score values have the same distribution on a significance level of 5%.

For evaluation of the phenotypic plasticity within the individuals of each population, we used d-values estimated using one-way multivariate analysis of variance (MANOVA) in combination with Bartlett test, using *manova1*-function in Matlab on the PCA score values of the first to tenth PC. Here, the d-values estimate the dimensionality of potential genotype-based multivariate group means by giving a value between 0 and *N-1*, with *N* being the amount of genotypes within the data. For most of the populations pollen from all four clones exist and were used for the estimation of d. For *A. odoratum*, Greece, one genotype contains the pollen grains of three different clones instead of four. In *A. odoratum*, Finland, for three different genotypes only the pollen grains of three clones are present. The pre-processing and analysis of the data were performed using Matlab R2016a (The Mathworks, Inc., Natick, MA, USA).

## Electronic supplementary material

Supplementary Information
