# Supplementary material for: Matrix-assisted laser desorption/ionization time-of-flight mass spectrometry (MALDI-TOF MS) shows adaptation of grass pollen composition
Source: Sci Rep. 2018 Nov 8;8:16591. doi: 10.1038/s41598-018-34800-1 (PMC6224550; doi:10.1038/s41598-018-34800-1)
Supplement: Supplementary file 1 — Supplementary Information [file 41598_2018_34800_MOESM1_ESM.pdf]

Supplementary Information

Matrix-assisted laser desorption/ionization time-of-flight mass spectrometry (MALDI-TOF MS) shows adaptation of grass pollen composition

Sabrina Diehn[1,2], Boris Zimmermann[3], Murat Bağcıoğlu[3], Stephan Seifert[1,2], Achim Kohler[3], Mikael Ohlson[4], Siri Fjellheim[5], Steffen Weidner[2] and Janina Kneipp[1,2]*

[1] Department of Chemistry, Humboldt-Universität zu Berlin, Brook-Taylor-Straße 2, 12489 Berlin, Germany

[2] BAM Federal Institute for Materials Research and Testing, Richard-Willstätter-Straße 11, 12489 Berlin, Germany

[3] Faculty of Science and Technology, Norwegian University of Life Sciences, 1432 Ås, Norway

[4] Faculty of Environmental Sciences and Natural Resource Management, Norwegian University of Life Sciences, 1432 Ås, Norway

[5] Faculty of Biosciences, Norwegian University of Life Sciences, 1432 Ås, Norway

* Corresponding author: janina.kneipp@chemie.hu-berlin.de.

Table S1: Classification results of the PLS models using full cross validation of pollen MALDI-TOF mass spectra using PLS with 9 latent variables for the classification of seven different populations. The numbers in the cells indicate the amount of spectra classified as the respective output class.

Target class<br>Output class	*A. odoratum* France	*A. odoratum* Greece	*A. odoratum* Finland	*F. ovina* Sweden	*P. alpina* Sweden	*P. alpina* Italy	*P. alpina* Norway
A. odoratum, France	32	0	9	0	0	0	0
A. odoratum, Greece	2	35	0	0	0	0	0
A. odoratum, Finland	2	0	24	0	0	0	0
F. ovina, Sweden	0	0	0	48	0	0	0
P. alpina, Sweden	0	0	0	0	39	0	0
P. alpina, Italy	0	0	0	0	1	40	0
P. alpina, Norway	0	0	0	0	0	0	40
Sucess Rate	89 %	100 %	73 %	100 %	98 %	100 %	100 %
